# Microwave-Assisted and Efficient Solvent-Free Knoevenagel Condensation. A Sustainable Protocol Using Porous Calcium Hydroxyapatite as Catalyst

**DOI:** 10.3390/molecules15020813

**Published:** 2010-02-04

**Authors:** Siham Mallouk, Khalid Bougrin, Abdelaziz Laghzizil, Rachid Benhida

**Affiliations:** 1 Laboratoire de Chimie des Plantes et de Synthèse Organique et Bioorganique, Université Mohammed V-Agdal, Faculté des Sciences, B.P. 1014 R.P, Rabat, Maroc; 2 Laboratoire de Chimie Physique Générale, Université Mohammed V-Agdal, Faculté des Sciences, B.P. 1014 R.P, Rabat, Maroc; 3 Laboratoire de Chimie des Molécules Bioactives et des Arômes, UMR 6001 CNRS, Institut de Chimie de Nice, Université de Nice-Sophia Antipolis, 28 avenue de Valrose, 06108 Nice Cedex 2, France

**Keywords:** solvent-free, sustainable procedure, microwave, Knoevenagel, catalysis

## Abstract

A sustainable Knoevenagel condensation of a series of aldehydes with malononitrile and ethyl cyanoacetate is described. The process is based on the combination of microwave activation and hydroxyapatite catalysis under solvent-free conditions. Products are obtained in and high yields after short reaction times. The effects of the specific surface of porous calcium hydroxyapatite and microwave activation are discussed.

## 1. Introduction

The Knoevenagel condensation is one of the most important, useful and widely employed methods for the formation of carbon-carbon bonds [1,2]. It has been used for the preparation of a range of substituted alkenes and bioactive molecules, and as a key step in natural product synthesis. In general, the Knoevenagel condensation is carried out using organic bases such as aliphatic amines, urea, ethylenediamine and piperidine or their corresponding ammonium salts, and the amino acids glycine or β-alanine [3,4]. Much work has shown that this condensation is strongly solvent-dependent and benzene, ethanol and DMF are commonly used. Other Lewis bases and acids have also been reported as catalysts in the Knoevenagel condensation, including ZnCl_2_ [5], CdI_2_ [6], TiCl_4_ [7], Al_2_O_3_ [8], Ni-SiO_2_ [9], MgO and ZnO [10], AlPO_4_–Al_2_O_3_ [11], KF–Al_2_O_3_ [12], natural phosphate [(NP)/KF or NP/NaNO_3_] [13] and synthetic phosphate (Na_2_CaP_2_O_7_ [14], Ca_2_P_2_O_7_ [14] and K_2_NiP_2_O_7_ [15]). However, the use of such bases/acids and solvents in large scale Knoevenagel reactions has led to environmental problems, *i.e.* the necessity to dispose of huge amounts of organic waste due to the formation of undesirable side products resulting from polymerisation, bis-addition and self-condensation; in addition to the total dissolved salts formed during the neutralisation of the base or acid catalysts. As a result other modified inorganic solids [16,17,18], resins [19], phase-transfer agents [20], ionic liquid [21,22], LDH-F hydrotalcite [23], cation-exchanged zeolites [16], or mesoporous materials [24], and MCM-41 [25] have been introduced as new catalysts. Electrochemical, microwave and ultrasound activation methods have also been reported [26,27,28,29,30,31]. Nevertheless, most of these known methods suffer from significant limitations, which include toxic reagents, harsh reaction conditions, low yields or long reaction times. Only a few sustainable procedures and particularly with basic heterogeneous catalyst were reported [32,33,34,35].

The application of MW irradiation as a non-conventional energy source for the activation of reactions has now become a very popular and useful technology in organic chemistry [36,37,38,39]. Moreover, the combination of MW activation and solvent-free conditions leads to enhanced conversion rates, higher yields, easier work-up and in general cleaner reactions confirming therefore the real advantages of this approach in the framework of green chemistry.

In continuation of our studies directed at the development of practical and efficient chemical processes [40,41,42,43], we report herein a sustainable Knoevenagel condensation protocol using a cooperative effect of MW activation and natural hydroxyapatite catalysis [Ca_10_(PO_4_)_6_(OH)_2_] (*p*-HAP) under solvent-free conditions. This strategy was applied for the condensation of malonitrile and ethyl cyanoacetate with different aldehydes (Table 1). The effect of MW and specific surface area of the catalyst on the rate enhancement are also dicussed.

## 2. Results and Discussion

First, to survey of the reaction conditions we chose malonitrile, ethyl cyanoacetate and aromatic aldehyde as model reaction partners (Table 1). The porous calcium hydroxyapatites used in our work—*p*-HAP100, *p*-HAP300 and *p*-HAP800—were prepared by a chemical wet method and heated at different temperatures (100, 300 and 800 °C, respectively) as reported in our previous work [44]. The mineral reagent thus obtained was impregnated with a 1:2 molar mixture of aldehyde **1** and ethyl cyanoacetate (**2a**) or malonitrile (**2b**), respectively. The reaction vessel was placed and irradiated in microwave reactor for 2 min.

Several parameters were surveyed to achieve optimum conditions, including stoichiometry of the reagents, irradiation time and microwave power levels. The best result was obtained when using 1 mmol of **1**, 1.2 mmol of **2a** or **2b** and 0.1 g/mmol of the *p*-HAP catalyst. For example, treatment of 4-nitrobenzaldehyde (**1b**) with 1.2 mmol of ethyl cyanoacetate (**2a**) in the presence of 0.1 g of *p*-HAP300 afforded alkene **3b** in almost quantitative yield (Table 1, entry 4). Under MW activation and *p*-HAP300 catalysis, all reactions proceeded efficiently, within short reaction times (Table 1). Products **3a-r** were isolated by simple filtration and were identified as the corresponding *E-*isomers. Their spectral data were identical to those reported in the literature (see references in Table 1). Both electron-rich and electron-poor benzaldehydes were converted to their corresponding alkenes in good to excellent yields, which indicated that the electronic effect did not significantly affect the reactivity (Table 1, entries 1–10, 12, 13–16, 18–19). Furancarboxaldehyde also undergoes clean condensation (Table 1, entries 11, 17). Moreover, aliphatic aldehydes showed good reactivity with **2a** and **2b** and led to products **3o**-**3r** in 89–98% yields (Table 1, entries 20–23). However, in the first condensation reaction, benzaldehyde gave only 46% yield of product **3a** (Table 1, entry 1). This result was clearly ascribed to the high sensitivity of benzaldehyde to air oxidation during the reaction. Indeed, when the reaction was carried out under nitrogen (Table 1, entry 2) or in the presence of hydroquinone as antioxidant (Table 1, entry 3) it provided **3a** in ca. 89% yield.

The use of microwave heating was critical to the success of the reaction. Indeed, control experiments conducted on 4-nitro- and 4-chlorobenzaldehyde (Table 1, entries 13, 14) showed that when the condensation was carried out under microwave irradiation, compounds **3h** and **3i** were obtained in ca. 95% yield, whereas only low yields (36% and 28% respectively, Table 1, entries 13, 14) were obtained under conventional heating due to the formation of side non-identified products as attested by TLC. The worst results obtained under conventional heating could be ascribed to the low thermal conductivity of *p*-HAP300 [40].

The specific microwave effects in this Knoevenagel condensation were then investigated in all reactions. For example, in Table 1 entry 4, a control experiment was conducted under the same reaction conditions: 4-nitrobenzaldehyde (**1a**) and *p*-HAP300 was reacted in a preheated oil bath at 76 °C for 1 h to 24 h, without solvent. Under these conditions the yield of product **3b** did not exceed 10% (GC-MS analysis), whereas under MW irradiation the same reaction led to a 96% yield. This result clearly attested to a significant specific non-thermal microwave effect, which gave rise to dramatic enhancement of the reactivity [45].

**Figure 1:**
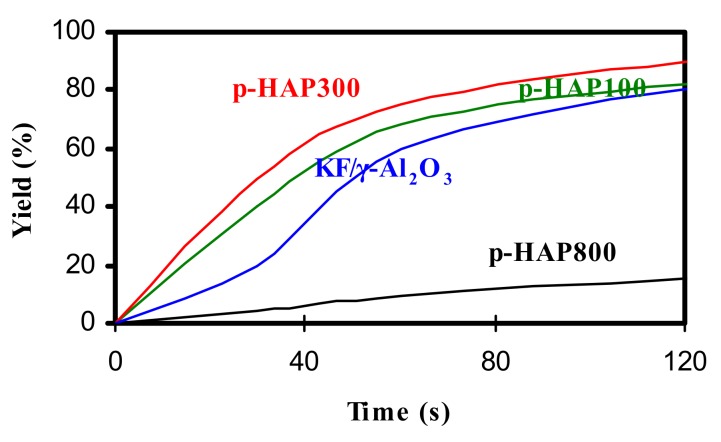
Catalytic activity of *p*-HAP100, *p*-HAP300, *p*-HAP800 and KF/Al_2_O_3_ in the Knoevenagel condensation of 4-nitrobenzaldehyde (**1b**) with ethyl cyanoacetate (**2a**).

We also compared the effect of the catalyst and used *p*-HAP100, *p*-HAP300 and *p*-HAP800 apatites and the known KF/Al_2_O_3_ hyperbase [28]. In a model reaction involving 4-nitrobenzaldehyde (**1b**) and ethyl cyanoacetate (**2a**), *p*-HAP100, *p*-HAP300 and KF-Al_2_O_3_ showed similar catalytic activity and efficiency after 2 h, with the best results being obtained with *p*-HAP300 (Figure 1). In contrast, *p*-HAP800 was found to be less effective. In line with this result, we observed that the yield of the reaction and the activity of the catalyst were affected by two major factors, *i.e.,* the effect of specific surface area and the chemical nature of the solid surface, as shown in Table 2.

**Figure 2:**
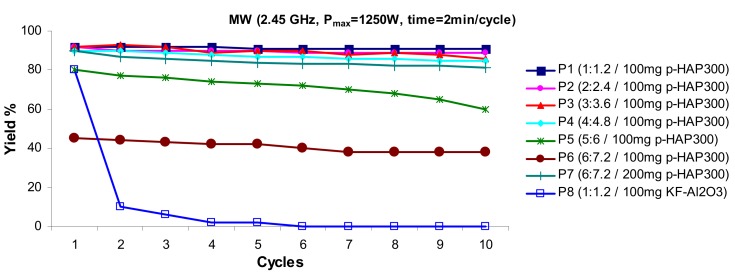
Recycling of the *p*-HAP300 catalyst for ten successive cycles and effect of molar ratio n(**1b)**:n(**2a**) on the yield of Knoevenagel condensation affording **3b**, under microwave irradiation.

Furthermore, the use of *p*-HAP300 is particularly convenient since this catalyst is efficiently recycled by simple heating at 300 °C under reduced pressure during 20 min. Excellent preservation of basic capacity of the *p*-HAP300 catalyst was observed. The good yields obtained during ten cycles (see Plots P1 to P4 in Figure 2) clearly attested for the preservation of the basicity and the high recyclability of this catalyst. The best yields were obtained with the optimum molar ratio of **1b**:**2a** = 1:1.2. The increase of this ratio decreased the yield (Figure 2, P5 and P6), which is probably due to the accumulation of the reactants on *p*-HAP300 surface. The use of an increased amount of *p*-HAP300 did not have a significant effect on the reaction yield (Figure 2, P7). In the case of KF-Al_2_O_3_ we found that the catalytic activity dramatically decreased after only one cycle (Figure 2, P8). In line with this result, previous work reported that in the absence of chemisorbed water and under microwave heating KF/Al_2_O_3_ did not catalyze the basic reaction after one cycle [46].

**Scheme 2:**
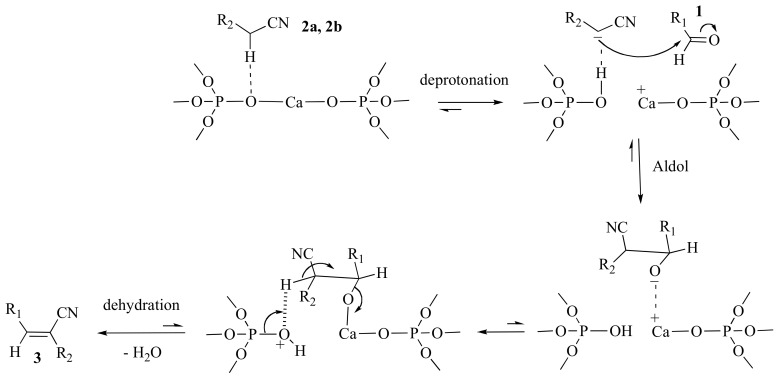
Plausible catalytic pathway for the *p*-HAP300-mediated Knoevenagel condensation under MW activation.

All together these results indicate that *p*-HAP300 is an excellent catalyst for the Knoevenagel condensation. By applying our procedure, the separation of the catalyst from the product only required simple filtration and the recyclability of the catalyst was higher compared to the conventional heating method. The high catalytic activity of *p*-HAP300 could be ascribed to the Ca^2+^–O^2−^ acid-base pair sites on the apatite surface that participate efficiently in the catalysis of deprotonation-aldol-dehydration steps, as proposed in Scheme 2.

## 3. Experimental

### 3.1. General

All reactions were run in dried glassware. Solvents were dried and distilled by standard procedures. Reagents were purchased (Alfa Aesar or Sigma-Aldrich) and used without further purification. All reactions were monitored by thin layer chromatography (TLC) plates (0.2 mm, silica gel 60). Flash chromatography was performed using silica gel (60, 0.040–0.063 mm). ^1^H NMR and ^13^C NMR were recorded on 200 and 500 instruments (200 and 500 MHz for ^1^H, 50 and 125 MHz for ^13^C). Chemical shifts (δ) were reported in parts per million and the coupling constants were reported in hertz (Hz). Reactions were irradiated in a multimode microwave oven (frequency: 2.45 GHz; maximal power: 1.250 kW).

### 3.2. Preparation of p-HAP100/300 and 800

The porous hydroxyapatite (denoted as *p*-HAp) was synthesized following a modified chemical wet method reported elsewhere [44]. At 25 °C, Ca(OH)_2_ (0.5M) was first dissolved in an ethanol-water mixture (1:1 volume ratio) and stirred for 3 hours. Aqueous solution of NH_4_H_2_PO_4_ (0.6M) was then added to the Ca(OH)_2_ solution over a period of 24 h. The amount of reagents in the solution were calculated to obtain a Ca/P molar ratio equals to 1.67, corresponding to a stoichiometric hydroxyapatite. The pH of the slurry was measured during the precipitation reaction at 25 °C, reaching a final value of ca. pH = 11. The precipitated crystals was aged for 24 h, filtered and dried at 100 °C overnight. The obtained powder is calcined at 300 and 800 °C for 3 hours. The data of these solids are described in our previous work [26].

The resulting *p*-HAP solids were characterized using X-ray powder diffraction (Philips PW131 diffractometer). The averge crystallite size of the precipitates was estimated by using the simple Scherrer Equation D = Kλ/(β_1/2_ cosθ) where D is the size (in Ǻ), K the shape factor equals to 0.9, λ the wavelength of X-rays equals to 1.5418 Ǻ, θ the diffraction angle for each reflexion and β_1/2_ is defined as the the diffraction peak width at half height (in radian). The particule size of the powders was analyzed by Dynamic Light Scattering on a Zetaplus (Broohaven Instruments) apparatus by suspending the powder in water by sonication. Infrared spectra were recorded at a 2 cm^−1^ resolution from 4000 to 400 cm^−1^ on a Bruker IFS 66v Fourier transform spectrometer using KBr pellets. The phosphorus content was assayed by absorption photometry after formation of the yellow phosphato-molybdic complex (*λ*_max_ = 460 nm). The calcium content was determined by complexometric titration with EDTA. Nitrogen adsorption isotherms were recorded at 77 K using a Micromeritics ASAP 2010 instrument. The specific surface areas (SSA) were calculated according to the Brunauer–Emmett–Teller (BET) method using adsorption data in the relative pressure range from 0.05 to 0.25 whereas the pore size and volume were estimated using the Barret-Joyner-Halenda (BJH) approximation.

### 3.3. Typical Procedure for the Knoevenagel Condensation

A mixture of malonitrile (**2b**, 79.3 mg; 1.2 mmol) and 4-nitrobenzaldehyde (**1a**, 151.1 mg, 1 mmol) was added to *p*-HAP300 (100 mg) in dichloromethane (5 mL). The solvent was evaporated under reduced pressure at moderate temperature (28 °C) and then the mixture was irradiated in a multimode microwave oven (frequency: 2.45 GHz; maximal power: 1.250 kW) for a determined period of time and temperature (see Table 1). The reaction progress was followed by TLC using *n*-hexane/ ethyl acetate (8:2) as eluent. After 2 min, the reaction vessel was cooled and the product was washed with dichloromethane or ethyl acetate (2 × 10 mL). The extract was filtered on a thin Celite bed and evaporated under reduced pressure to leave a pale yellow solid, which was recrystallized from ethanol to give pure 4-nitrobenzylidenemalonitrile (**3h**, 192 mg, 96%) as pale yellow crystals, mp = 160–162 °C (Lit. [[15a] 160 °C) ^1^H-NMR (CDCl_3_): δ 7.78 (s, 1H, CH=), 8.09 (d, 2H, *J* = 8.9 Hz, ArH), 8.38 (d, 2H, *J* = 8.9 Hz, ArH); IR (KBr): ν 3035, 2228, 1579, 1522 cm^-1^; EI-MS: m/z (%):199 (M^+^), 142; Anal. Calcd for C_12_H_10_N_2_O_4_: C, 58.54, H, 4.09; Found: C, 58.65, H, 4.21.

## 4. Conclusions

In summary, we have reported an efficient and sustainable solvent-free Knoevenagel condensation using a cooperative effect of MW activation and *p*-HAP300 catalysis. The procedure is convenient and highly efficient since the title compounds are produced in good to excellent yields after short reaction times. Moreover, *p*-HAP300 showed high thermal stability and can be recovered by simple filtration and reused for at least ten reaction cycles without loss of activity. Efforts to extend this green procedure to other functionnalised substrats including heteroaromatic aldehydes and ketones are under way.

## Figures and Tables

**Table 1:**
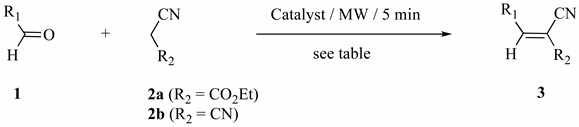
Knoevenagel condensation of aldehydes with ethyl cyanoacetate **2a** and malonitrile **2b** catalyzed by p-hydroxyapatite under solvent-free and microwave irradiation (Power = 1250 W, irradiation time = 2 min).

Entry	Product	R_1_	R_2_	Catalyst	T(°C)	Yield %	Mp (°C)[Lit.]
1	**3a**	C_6_H_5_	CO_2_Et	*p*-HAP300	85	46	52–54 (50) [21]
2 ^a^	**3a**	C_6_H_5_	CO_2_Et	*p*-HAP300/N_2_	80	89	
3 ^b^	**3a**	C_6_H_5_	CO_2_Et	*p*-HAP300/ hydroquinone	88	88	
4	**3b**	4-NO_2_C_6_H_4_	CO_2_Et	*p*-HAP300	76	96 (10)^d^	170–172 (172–173) [28]
5	**3b**	4-NO_2_C_6_H_4_	CO_2_Et	*p*-HAP100	62	82	
6	**3b**	4-NO_2_C_6_H_4_	CO_2_Et	*p*-HAP800	50	15	
7	**3b**	4-NO_2_C_6_H_4_	CO_2_Et	KF/Al_2_O_3_	88	80	
8	**3c**	4-ClC_6_H_4_	CN	*p*-HAP300	57	93	162–164 (162) [21]
9	**3d**	4-CH_3_OC_6_H_4_	CN	*p*-HAP300	78	92	115–117 (116) [21]
10	**3e**	4-CH_3_C_6_H_4_	CN	*p*-HAP300	54	91	125–127 (129) [21]
11	**3f**	2-furyl	CN	*p*-HAP300	92	89	87–89 (91–92) [14]
12^b^	**3g**	C_6_H_5_	CN	*p*-HAP300/ hydroquinone	81	87	
13	**3h**	4-NO_2_C_6_H_4_	CN	*p*-HAP300	56	96 (36)^d^	160–162 (161–162) [29]
14	**3i**	4-ClC_6_H_4_	CO_2_Et	*p*-HAP300	70	95 (28)^d^	92–94 (91) [21]
15	**3j**	4-CH_3_OC_6_H_4_	CO_2_Et	*p*-HAP300	64	94	86–88 (83–84) [28]
16	**3k**	4-CH_3_C_6_H_4_	CO_2_Et	*p*-HAP300	81	96	90–92 (94–95) [28]
17	**3l**	2-furyl	CO_2_Et	*p*-HAP300	105	90	68–70 (72) [14]
18	**3m**	4-(CH_3_)_2_NC_6_H_4_	CO_2_Et	*p*-HAP300	89	87	125–127 (126–127) [28]
19	**3n**	4-HOC_6_H_4_	CO_2_Et	*p*-HAP300	101	80	170–172 (171–172) [28]
20	**3o^e^**	propyl	CO_2_Et	*p*-HAP300	87	89	Liquid [21]
21	**3p^e^**	methyl	CO_2_Et	*p*-HAP300	87	91	Liquid [21]
22	**3q^e^**	propyl	CN	*p*-HAP300	89	88	Liquid [21]
23	**3r^e^**	methyl	CN	*p*-HAP300	89	98	Liquid [21]

^a^ Air is excluded by purging the reaction vessel with nitrogen. ^b^ 20% molar of hydroquinone was added. ^c^ Yields of pure products obtained under MW irradiation. ^d^ Yields of pure products obtained under conventional heating. ^e^ Liquid products **3o-r** were purified by chromatography on silica gel (hexane/ethyl acetate: 9/1).

**Table 2:** The specific surface area effect of supports on Knoevenagel condensation involving 4 nitrobenzaldehyde (**1b**) and **2a** under microwave irradiation.

Support	Time (min)	T (°C)	Specific Surface Area (m^2^.g^−1^)	Yield (%)^a^
p-HAP100	2	62	253	82
p-HAP300	2	76	159	96
p-HAP800	2	50	89	15
KF/Al_2_O_3_	2	88	High	80

^a^ Isolated yield of pure **3b** obtained in the condensation of 4-nitrobenzaldehyde (1 mmol), malonitrile (1.2 mmol) and catalyst (100 mg) under solvent-free microwave irradiation.
